# Inhibition of GSK3β and RIP1K Attenuates Glial Scar Formation Induced by Ischemic Stroke *via* Reduction of Inflammatory Cytokine Production

**DOI:** 10.3389/fphar.2020.00812

**Published:** 2020-06-12

**Authors:** Jin Liu, Yong-Ming Zhu, Yi Guo, Liang Lin, Zhan-Xiang Wang, Feng Gu, Xin-Yi Dong, Ming Zhou, Yi-Fan Wang, Hui-Ling Zhang

**Affiliations:** ^1^Jiangsu Key Laboratory of Neuropsychiatric Diseases and College of Pharmaceutical Sciences, Department of Pharmacology and Laboratory of Cerebrovascular Pharmacology, College of Pharmaceutical Science, Jiangsu Key Laboratory of Preventive and Translational Medicine for Geriatric Diseases, School of Public Health, Soochow University, Suzhou, China; ^2^Department of Anesthesiology, Department of Neurosurgery, The First Affiliated Hospital of Xiamen University, Xiamen, China

**Keywords:** ischemic stroke, glial scar, astrocyte, glycogen synthase kinase-3β, receptor-interacting protein 1 kinase, inflammation

## Abstract

In the chronic phase following ischemic stroke, glial scars can prevent axonal regeneration and the intensification of inflammation. The protective effect of inhibition of glycogen synthase kinase-3β (GSK3β) or receptor-interacting protein 1 kinase (RIP1K) on ischemic stroke has been previously reported. The current study examined the effects of RIP1K and GSK3β on ischemic stroke-induced glial scar formation. To investigate this, we used an *in vivo* model of ischemic stroke based on middle cerebral artery occlusion for 90 min followed by reperfusion for 7 d, and an *in vitro* model in primary cultured astrocytes involving oxygen and glucose deprivation for 6 h followed by reoxygenation for 24 h. Both *in vivo* and *in vitro*, we found that SB216763, a GSK3β inhibitor, and necrostatin-1 (Nec-1), a RIP1K inhibitor, decreased levels of glial scar markers, including glial fibrillary acidic protein (GFAP), neurocan, and phosphacan. SB216763 and Nec-1 also decreased levels of inflammatory related cytokines, including interleukin-6 (IL-6), interleukin-1 β (IL-1β), and tumor necrosis factor-α (TNF-α). However, only Nec-1 increased the level of interleukin-1 receptor antagonist. Concurrent neutralization of TNF-α, IL-1β, and IL-6 with their antibodies provided better reduction in oxygen and glucose deprivation-induced increases in scar markers than obtained with separate use of each antibody. Further investigations showed that SB216763 reduced the levels of necroptosis-related proteins, including RIP1K, p-RIP1K, RIP3K, p-RIP3K, mixed lineage kinase domain-like protein (MLKL), and p-MLKL, while Nec-1 decreased the expression of p-GSK3β. Compared with Nec-1 (10 μM) and SB216763 (1 μM) alone, Nec-1 and SB216763 in combination reduced levels of GFAP, neurocan, and inflammatory-related cytokines. In conclusion, inhibition of GSK3β or RIP1K reduced glial scar formation induced by ischemic stroke. The underlying mechanisms might be at least, partially related to reducing levels of inflammatory-related cytokines and to blocking an interaction between GSK3β- and RIP1K-mediated pathways.

## Introduction

Ischemic stroke is the third most common cause of death in most Western countries (Durukan and Tatlisumak, 2007; Sommer, 2017) and has become the most common cause of death in China (Wang et al., 2017), usually resulting from an atherosclerotic thrombus formation in a major cerebral artery. To date, tissue plasminogen activator (tPA) is the only clinically effective drug approved by the Food and Drug Administration (FDA) for ischemic stroke (Zhao et al., 2017; Xu et al., 2018). However, only 20% of patients with ischemic stroke receive tPA treatment because of its limited therapeutic window of 4.5 h and its severe side effects, including an increased risk of symptomatic intracerebral hemorrhage (Deng et al., 2019). Therefore, to develop novel therapeutic drugs is still under evaluation.

Astrocytes are the most abundant glial cell type in the brain and play a key role in reactive astrogliosis and glial scar formation after ischemic stroke (Barreto et al., 2011; Sims and Yew, 2017). Reactive astrogliosis helps to protect brain cells against severe injury (Mohn and Koob, 2015) and to avoid oxidative stress in the acute stage. On the other hand, glial scar formation inhibits axonal growth and leads to the production of a large number of inflammatory cytokines in the chronic stage (Binder and Steinhauser, 2006; Pluchino et al., 2008; Yang et al., 2011; Burda and Sofroniew, 2014; Chen et al., 2016b). In the scar, reactive astrocytes express inhibitory molecules, including chondroitin sulfate proteoglycans (CSPGs) such as neurocan, phosphacan, and aggrecan, which inhibit axonal regeneration (Liu and Chopp, 2016). Therefore, exploring the molecular mechanisms underlying glial scar formation is critical for improving our understanding of ischemic stroke.

An increasing amount of evidence has emerged to suggest that neuroinflammatory process plays an important role in the pathophysiological process of cerebral ischemia/reperfusion (I/R) injury. Except that activation of resident brain cells (mainly microglia) and various types of inflammatory cells (including neutrophils, different subtypes of T cells, monocyte/macrophages, and other cells) infiltrated into the ischemic brain tissue are involved in the ischemia/reperfusion-induced inflammatory process (Jin et al., 2010), astrocytes also have a powerful pro-inflammatory effect, emerging as pivotal regulators of inflammatory responses in the central nervous system (Buffo et al., 2010; Sofroniew, 2015). Within minutes after injury, injured neurons and glial cells in the core and penumbra of the stroke produce pro-inﬂammatory mediators, including interleukin-6 (IL-6), interleukin-1 β (IL-1β), and tumor necrosis factor-α (TNF-α) (Cai et al., 2006; Shekhar et al., 2018), which activate both astrocytes and microglia (Tuttolomondo et al., 2008). Interleukin-1 receptor antagonist (IL-1Ra) is an endogenous antagonist of IL-1β which can limit the action of the cytokine IL-1β (Clausen et al., 2016). Therefore, the inflammatory cascade is an important target for limiting ischemic tissue damage and reducing glial scar formation.

Recently, glycogen synthase kinase-3β (GSK3β) was identified as a key regulator of the inflammatory response. GSK3β is a serine-threonine protein kinase which regulates numerous cell signaling pathways, including cell death and survival pathways (Klamer et al., 2010; Singh et al., 2015). Activation of GSK3β is correlated with phosphorylation at tyrosine 216, and phosphorylation within the amino-terminal domain of GSK3β (Ser 9) results in inactivation of GSK3β by several kinases including Akt (Ohori et al., 2008). Studies have reported that GSK3β inhibitor Ro3303544 is beneficial for spinal cord injury (SCI) as it inhibits glial scar formation (Renault-Mihara et al., 2011). Previous studies have revealed that GSK3β plays roles in the inflammatory response of microglia and astrocytes, showing that GSK-3β inhibitors suppressed inflammatory response in 6-OHDA-activated astrocytes or in autophagy deficiency microglia (Zhou et al., 2011; Wang et al., 2013). However, whether GSK3β inhibitor can block the ischemic stroke-induced formation of glial scar associated with suppressing astrocyte-mediated inflammation and its underlying mechanisms are largely unclear.

Necroptosis, a form of regulated necrotic cell death, is regulated by receptor interacting protein kinase 1 (RIP1K), which promotes the sequential activation of receptor interacting protein kinase 3 (RIP3K) and mixed lineage kinase domain-like protein (MLKL) (He et al., 2009; Sun et al., 2012; Kim et al., 2017). There is some evidence suggesting that necroptosis is involved in all stages of the ischemic cascade (Xu and Zhang, 2011). Previous results from our group and others have shown that necrostatin-1 (Nec-1), a specific inhibitor of RIP1K, improves transient and permanent focal cerebral ischemia-induced brain injuries in rats by inhibiting the activation of RIP1K or by blocking the formation of the RIP1K–RIP3K complex, oxidative stress, and the expression of inflammatory cytokines, including TNF-α, IL-1β, IL-2, and IL-6 (Rezaie and Dean, 2002; Xu et al., 2010; Li et al., 2018; Ni et al., 2018). Further, in zVAD.fmk-induced necroptosis of L929 cells or J774 cells, a mouse macrophage cell line, Nec-1 treatment could inhibit the production of TNF-α depending the kinase activity of RIP1 (Christofferson et al., 2012). These findings indicate that RIP1 kinase plays crucial roles in mediating necroptosis and inflammation in cellular models of necroptosis. However, whether RIP1 kinase inhibitor can block the ischemic stroke-induced formation of glial scar related to inhibiting astrocyte-mediated inflammation and its underlying mechanisms remains investigated. It has been found that RIP1K can activate the PI3K-Akt pathway, in which GSK3β is an important downstream protein (Park et al., 2008). Therefore, we raised the hypothesis that RIP1K and GSK3β may play a key role in ischemic stroke-induced astrogliosis and glial scar formation, and there might be an interaction between them.

In the present study, we found that both the GSK3β inhibitor SB216763 and the RIP1K inhibitor Nec-1 attenuate glial scar formation, at least, partially related to inhibiting astrocyte-mediated inflammatory response and to reducing the interaction of GSK3β and RIP1K. Our results suggest that GSK3β and RIP1K might be potential therapeutic targets for ischemic stroke.

## Materials and Methods

### Animals

Male Sprague-Dawley (SD) rats (weight, 280–310 g) were purchased from SLAC (Shanghai, China). All animal procedures and protocols used in this study were approved by the Soochow University Animal Care and Use Committee (Use license: SYXK-2016-0050; Production license: SYXK-2017-0006). Rats were kept in a temperature and humidity-controlled room with a 12 h/12 h light/dark cycle and free access to water.

### Transient Middle Cerebral Artery Occlusion Model

Rats were subjected to Transient Middle Cerebral Artery Occlusion (tMCAO) as previously described (Zhu et al., 2017). Briefly, after rats were anesthetized with isoflurane, the right common carotid artery, internal carotid artery, and external carotid artery were exposed through a midline cervical incision. Then, a 4-0 nylon suture was inserted into the internal carotid artery until it occluded the origin of the middle cerebral artery. For reperfusion, the suture was gently withdrawn 90 min after ischemia. Rats in the sham groups experienced the same operation except that there was no suture inserted. Rats were randomly assigned to groups using the online tool QuickCalcs (http://www.graphpad.com/quickcalcs/). Necrostatin-1 (Nec-1) at 48 nmol (#S8037; Selleck, shanghai, China), a RIP1K inhibitor, or SB216763 at 400 pmol (#S1075; Selleck, shanghai, China), a GSK3β inhibitor, dissolved in 1% DMSO, was administrated intracerebroventricularly 10 min before MCAO, respectively.

### Primary Cortical Astrocyte Culture and Oxygen-Glucose Deprivation/Reoxygenation

Primary astrocyte cultures were prepared from the cortex of SD rat brains at postnatal day 1 (P1), as previously described (Li et al., 2018). Briefly, after mechanical dissociation in phosphate-buffered saline (PBS), the cell suspension was incubated for 8 min at 37°C in 2.5% trypsin and dissociated in DNase (#D7073; Beyotime, Shanghai, China) solution. Cell suspension were filtered through a sterile 40-μm nylon cell strainer and was purified by the differential adhesion method and the shaking protocol, and then was seeded on poly-L-lysine (PLL, #P1399; Sigma-Aldrich, MO, USA) coated plates and grown at 37°C in a humidified atmosphere with 5% CO_2_/95% air. The medium for cell culture was Dulbecco’s Modified Eagle’s Medium (DMEM/F12 at 1:1, #11330; Gibco, MA, USA) containing 10% fetal bovine serum (#10099; Gibco, MA, USA) and 1% 100 U/ml penicillin/streptomycin (#C0222; Beyotime, shanghai, China).

Astrocytes were exposed to oxygen and glucose deprivation (OGD) and reoxygenation (OGD/Re) as previously described (Qin et al., 2019). Briefly, the culture medium was replaced with serum- and glucose-free DMEM (#11966; Gibco, MA, USA). Then astrocytes were placed in a humidified and sealed hypoxic chamber (Billups-Rothenberg, CA, USA), with 5% CO_2_ and 95% N_2_ for 6 h at 37°C. For reoxygenation, astrocytes were incubated in complete medium maintained with 5% CO_2_ atmosphere at 37°C. Nec-1 or SB216763 was dissolved in DMSO to obtain a 100 or 10 mM stock solution, respectively. SB216763 at 1 or 5 uM and Nec-1 at 10 or 100 uM were added to the cells starting from OGD and from reoxygenation, respectively.

In order to observe the effect of neutralization of TNF-α, IL-1β, and IL-6 with their antibodies on astrocyte scar, TNF-a antibody (Neut, 10 μg/ml; #ab7742; Abcam, Cambridge, UK), IL-1β antibody (Neut, 10 μg/ml; #GTX31180; GeneTex, CA, USA), or IL-6 antibody (Neut, 10 μg/ml; #GTX59838; GeneTex, CA, USA) was added to the cells starting from reoxygenation for 24 h.

### Western Blotting Analysis

Isolated brain cortices or cultured astrocytes were washed three times in ice-cold PBS and lysed in lysis buffer (10 mM Tris HCl, 150 mM NaCl, 1% TritonX-100, 1% sodium deoxycholate, 0.1% sodium dodecyl sulfate, 5 mM EDTA; pH 7.4) containing phosphatase inhibitors and a protease. Nitrocellulose membranes or PVDF membranes were incubated with specified primary antibodies (**Table 1**) at 4°C overnight. This was followed by incubation at room temperature for 1 h with corresponding secondary antibodies (**Table 2**). Immunoreactivity was detected and blots were captured with an Odyssey scanner (LI-COR Biosciences, NE, USA). Densitometry analyses of bands were performed using Fiji-ImageJ (https://imagej.net/Fij), with signal intensity values normalized to β-actin.

**Table 1:** Information for primary antibodies used.

Protein	Usage	Antibody	Brand
TNF-a	Neut (10 μg/ml)	#ab7742	Abcam, Cambridge, UK
IL-1β	Neut (10 μg/ml)	#GTX31180	GeneTex, CA, USA
IL-6	Neut (10 μg/ml)	#GTX59838	GeneTex, CA, USA
GFAP	WB (1:2,000)	#MAB360	Millipore, MO, USA
GFAP	IF (1:1,000), IHC (1:500)	#ab4674	Abcam, Cambridge, UK
GFAP	IF (1:500), IHC (1:500)	#C9205	Sigma-Aldrich, MO, USA
IL-6	WB (1:500)	#NB600-1131	Novus, CO, USA
IL-1β	WB (1:500)	#ab9722	Abcam, Cambridge, UK
IL-1β	WB (1:1,000)	#GTX74034	GeneTex, CA, USA
TNF-α	WB (1:1,000)	#ab6671	Abcam, Cambridge, UK
GSK3β	WB (1:1,000)	#9315	Cell Signaling Technology, MA, USA
Phospho-GSK3β	WB (1:1,000), IF (1:100)	#9323	Cell Signaling Technology, MA, USA
IL-1Ra	WB (1:5,000)	#ab124962	Abcam, Cambridge, UK
RIP1K	WB (1:200)	#610458	BD Transduction, MD, USA
Phospho-RIP1K	WB (1:1,000)	#31122	Cell Signaling Technology, MA, USA
RIP3K	WB (1:1,000)	#ab62344	Abcam, Cambridge, UK
Phospho-RIP3K	WB (1:1,000)	#ab195117	Abcam, Cambridge, UK
MLKL	WB (1:1,000)	#orb32399	Biorbyt, Cambridge, UK
MLKL	WB (1:1,000)	#MABC604;	Millipore
Phospho-MLKL	WB (1:1,000)	#ab196436	Abcam, Cambridge, UK
Phosphacan	WB(1:500), IF (1:500), IHC (1:200)	#P8874	Sigma-Aldrich, MO, USA
Neurocan	WB (1:500), IF (1:200), IHC (1:200)	#ab26003	Abcam, Cambridge, UK
β-actin	WB (1:5,000)	#A5441	Sigma-Aldrich, MO, USA

**Table 2:** Information for secondary antibodies used.

Protein	Usage	Antibody	Brand
Anti-mouse IgG (H + L)	WB (1:10,000)	#042-06-18-06	KPL, MA, USA
Anti-rabbit IgG (H + L)	WB (1:10,000)	#042-06-15-06	KPL, MA, USA
Alexa Fluor^®^ 488 goat Anti-rabbit IgG (H + L)	IF (1:500), IHC (1:500)	#A11008	Life Technologies, MA, USA
Alexa Fluor^®^ 488 goat Anti-chicken IgG (H+ L)	IF (1:1,000)	#ab150169	Abcam, Cambridge, UK
Alexa Fluor^®^ 594 goat Anti-mouse IgG (H + L)	IF (1:500), IHC (1:500), IHC (1:500)	#A11005	Life Technologies MA, USA

### Immunocytochemistry

Astrocytes were seeded on 24-well plates containing PLL-coated coverslips and fixed with 4% paraformaldehyde for 10 min. They were then washed in PBS, permeabilized with 0.1% TritonX-100 for 30 min, and blocked with 1% bovine serum albumin (BSA) for 60 min at room temperature. Finally, cells were incubated at 4°C overnight with the specified primary antibodies (**Table 1**). Detection of each labeled primary antibody was performed by incubation for 1 h at room temperature with the corresponding fluorescent secondary antibody (**Table 2**). Nuclei were labeled with Hoechst stain (1:5,000; #33258; Sigma-Aldrich, MO, USA). Images were obtained using a laser confocal microscope (LSM 710; Carl Zeiss, Oberkochen, Germany) or a fluorescence microscope (TE-2000; Nikon, Tokyo, Japan) and the exposure times were kept the same for all images taken. The fluorescence intensity was determined over the GFAP^+^ cell using build-in and custom-written ImageJ plugins and normalized to the background.

### Immunohistochemistry

Immunohistochemistry was performed as previously described (Qin et al., 2019). At 7 d after MCAO, the brains were perfused and fixed with 4% paraformaldehyde for 24 h, dehydrated by increasing concentrations of saccharose, embedded in OCT (optimal cutting temperature compound, Sakura, USA), and then cut into 12-μm thick sections in the coronal plane. Subsequently, brain sections were fixed with 4% paraformaldehyde for 10 min, permeabilized with 0.3% Triton X-100, and blocked by 1% BSA for 1 h at room temperature, and incubated with specific primary antibodies (**Table 1**) overnight at 4°C and corresponding secondary antibodies (**Table 1**) for 1 h at room temperature. Then, the sections were incubated for 20 min with Hoechst solution to stain nuclei. Images were obtained by a confocal laser scanning microscopy (LSM 710, Carl Zeiss, Germany) and the exposure times were kept the same for all images taken. Fluorescence intensity was determined using build-in and custom-written ImageJ plugins and normalized to the background. Results are expressed as mean fluorescence intensity (fluorescence intensity per unit area, AU./μm^2^).

### Cell Death Analysis

#### Propidium Iodide Staining

After OGD/Re treatment, astrocytes were incubated with propidium iodide (PI) (5 μg/ml; #P4170; Sigma-Aldrich, MO, USA) and nuclei were labeled with Hoechst (1:5,000; #33258; Sigma-Aldrich, MO, USA) for 20 min. Images were obtained using a laser confocal microscope (LSM 710; Carl Zeiss, Jena, Germany).

#### Lactate Dehydrogenase Leakage Measurement

Cell viability was measured at 450 nm with an automatic multiwall spectrophotometer (Bio-Rad Laboratories, Hercules, CA, USA) using lactate dehydrogenase (LDH) (Nanjing Jiancheng Bioengineering Institute, Nanjing, China), as previously described (Qin et al., 2019). Briefly, after treatment, the medium was collected and placed on ice, and astrocytes were lysed with 1% TritonX-100 at 37°C for 30 min. Samples of media and cell lysates were prepared according to the manufacturer’s protocol. LDH leakage was calculated as follows: LDH leakage (%) = OD value of the supernatant of the medium/(OD value of the supernatant of the medium + OD value of the supernatant of lysed cells) × 100%.

### Astrocyte Proliferation

Astrocyte proliferation was determined using the Cell-Light EdU (5-ethynyl-2′-deoxyuridine) Apollo567 In Vitro Kit (#C10310; Riobio, Guangzhou, China) following the manufacturer’s instructions. SB216763 or Nec-1 were added to the medium during reoxygenation and the astrocytes were incubated for 24 h. Cells were sequentially incubated with 20 μM EdU during reoxygenation; 4% paraformaldehyde (to fix the cells for morphological analysis) for 30 min at room temperature; and 0.5% Triton X-100 (to permeabilize the cells) for 10 min. Finally, 1× Apollo reaction solution was added and the cells were incubated for 30 min. To label GFAP, cells were exposed overnight to an anti-GFAP antibody (1:1,000, #53-9892-82, Thermo, MA, USA) at 4°C. Hoechst (1:5,000; #33258; Sigma-Aldrich, MO, USA) was used to stain the nuclei. Images were obtained using a confocal microscope (LSM 710; Carl Zeiss, Jena, Germany).

### Statistical Analysis

All data were estimated as the mean value ± SD. Differences were compared using a one-way analysis of variance (ANOVA), followed by Tukey’s *post hoc* test. P < 0.05 was considered statistically significant.

## Results

### SB216763 Protects Against OGD/Re-Induced Astrocytic Cell Death

**Figure 1:**
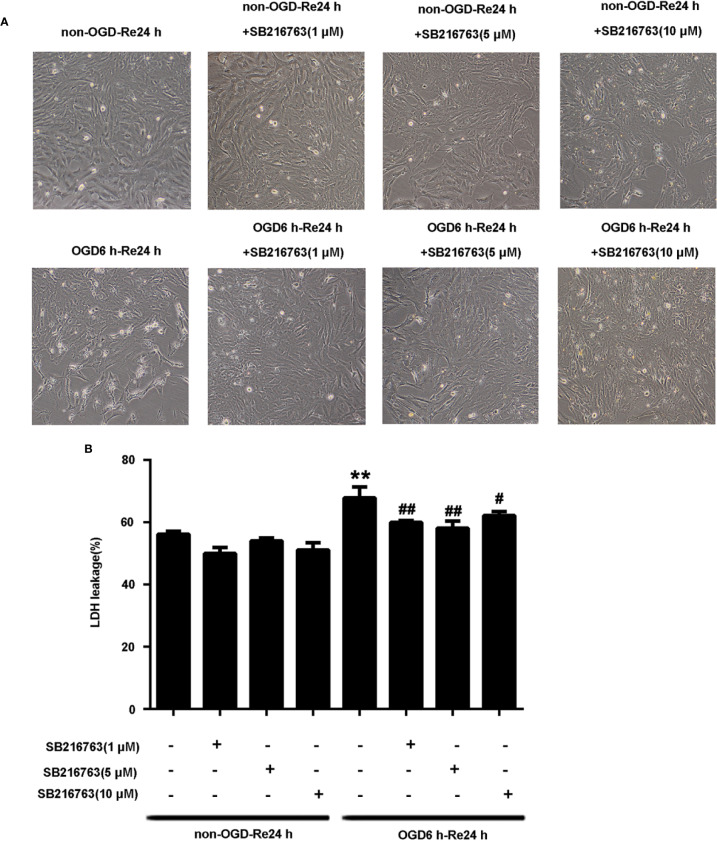
SB216763 protects astrocytes from oxygen and glucose deprivation (OGD)/re-induced cell injury. **(A)** Representative light microscopy images of astrocytes exposed to OGD for 6 h and reoxygenation for 24 h. Astrocytes were treated with different concentrations of SB216763 during OGD and reoxygenation. **(B)** Columns present data from the quantitative analysis of lactate dehydrogenase leakage in panel A. Mean ± SD, n = 3. ^**^P < 0.001 vs. non-OGD-Re24 h group; ^#^*P* < 0.05, ^##^*P* < 0.01 vs. OGD6 h-Re24 h group.

The protective effect of GSK3β inhibitor SB216763 following OGD/Re injury has been reported previously for neurons (Chen et al., 2016a), but not for astrocytes following OGD/re-induced injury. Therefore, we established the OGD/Re model *in vitro* to mimic *in vivo* I/R injury, by applying SB216763 during OGD/Re and observing its effect on astrocytic cell death. LDH results showed that application of SB216763 at 1, 5, or 10 μM protected astrocytes following OGD/reinjury, as shown by the reduction of LDH leakage (**Figure 1**). The 5 μM dose showed the strongest protective effect (**Figure 1**). Therefore, 5 μM was chosen as the optimum concentration for the application of SB216763 *in vitro* in the following experiments.

### SB216763 Reduces Ischemic Stroke-Induced Astrogliosis *In Vivo* and *In Vitro*

**Figure 2:**
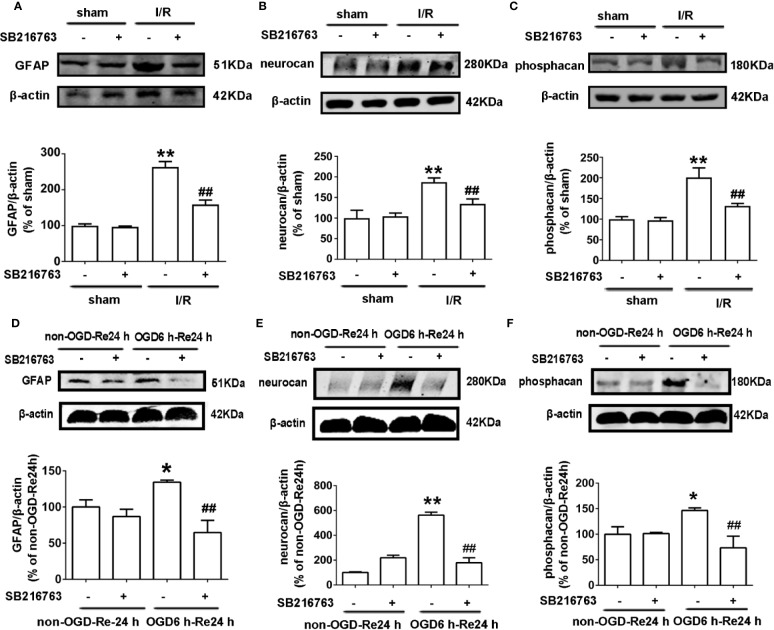
SB216763 reduces the expression of glial scar-related proteins after oxygen and glucose deprivation (OGD) for 6 h and reoxygenation for 24 h, and after ischemia/reperfusion (I/R) injury in rats. **(A–C)** Representative images from Western blotting (WB) analysis of the levels of GFAP, neurocan, phosphacan in the peri-infarct region of sham or cerebral ischemic cortex at 7 d after reperfusion following ischemia for 90 min. Columns present data from the quantitative analysis of GFAP, neurocan, and phosphacan immunoblots, respectively. SB216763 (400 pmol) was intracerebroventricularly administrated before ischemia. β-Actin protein was used as a loading control. Mean ± SD, n = 3. ^**^P < 0.01, ^*^P < 0.05 vs. sham group; ^##^*P* < 0.01, ^#^*P* < 0.05 vs. I/R group. **(D–F)** Representative images from WB analysis of the levels of glial fibrillary acidic protein (GFAP), neurocan, phosphacan under conditions of OGD for 6 h, and reoxygenation for 24 h. The order of columns and loading control used are the same as in panels A–C. Astrocytes were exposed to OGD for 6 h and reoxygenation for 24 h. Astrocytes were treated with SB216763 (5 μM) during OGD and reoxygenation. Mean ± SD, n = 3. ^*^P < 0.05, ^**^P < 0.01 vs. non-OGD-Re24 h group; ^##^*P* < 0.01 vs. OGD6 h-Re24 h group.

**Figure 3:**
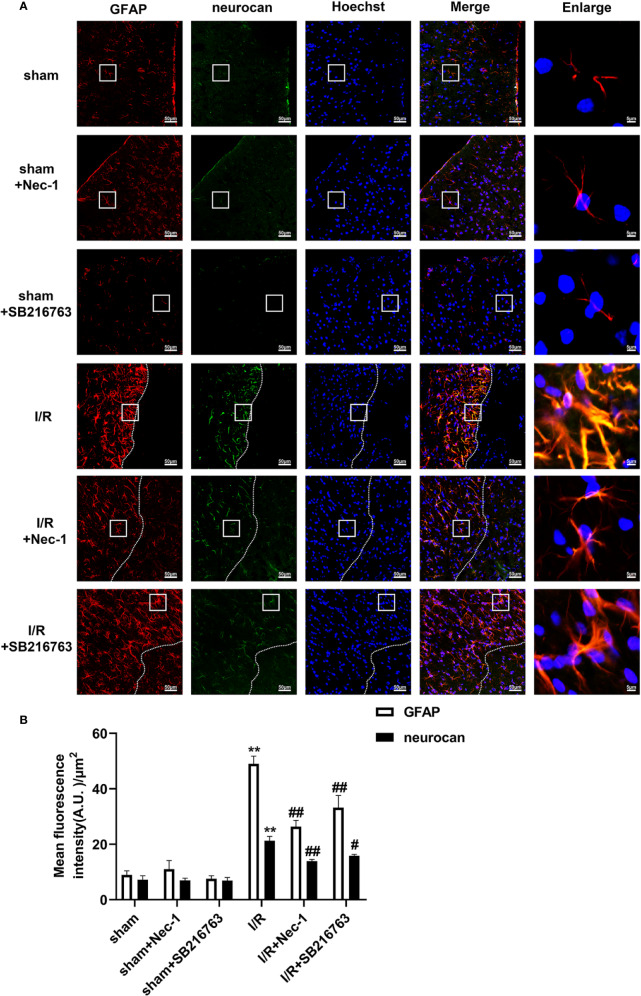
SB216763 and Nec-1 reduces the fluorescence intensity of glial fibrillary acidic protein (GFAP) and neurocan in astrocytes after ischemia/reperfusion (I/R) in rats. SB216763 (400 pmol) or Nec1 (48 nmol) was intracerebroventricularly administered before ischemia. **(A)** Representative images of GFAP, neurocan, and Hoechst staining in the peri-infarct zones of the sham or cerebral ischemic cortex at 7 d after reperfusion following tMCAO for 90 min (GFAP: red; neurocan: green; Hoechst: blue). The white dotted line represents the edge between the infarct area and the peri-infarct zones, and the white boxes indicate the corresponding area of the enlarged images shown below. **(B)** Quantification of fluorescence intensity of GFAP and neurocan in panel A. Mean ± SD, n = 3. ^**^P < 0.01 vs. sham group; ^#^*P* < 0.05, ^##^*P* < 0.01 vs. I/R group.

**Figure 4:**
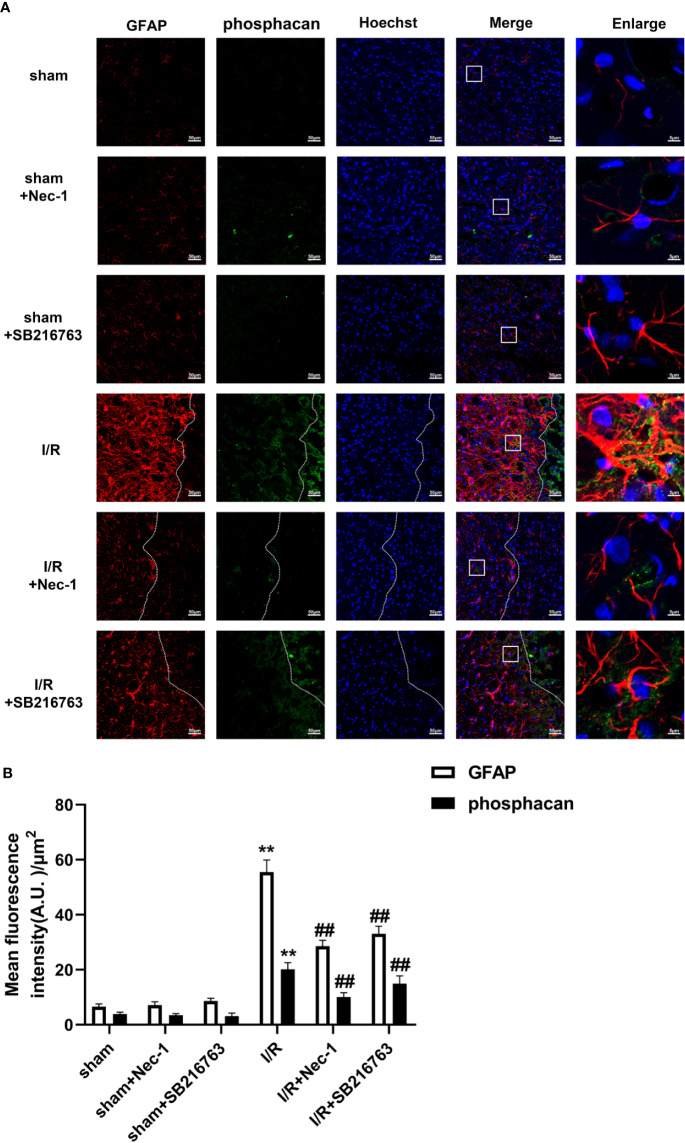
SB216763 and Nec-1 reduces the fluorescence intensity of glial fibrillary acidic protein (GFAP) and phosphacan in astrocytes after ischemia/reperfusion (I/R). SB216763 (400 pmol) or Nec1 (48 nmol) was intracerebroventricularly administered before ischemia. **(A)** Representative images of GFAP, phosphacan, and Hoechst staining in the peri-infarct zones of the sham or cerebral ischemic cortex at 7 d after reperfusion following tMCAO for 90 min (GFAP: red; phosphacan: green; Hoechst: blue). The white dotted line represents the edge between the infarct area and the peri-infarct zones, and the white boxes indicate the corresponding area of the enlarged images shown below. **(B)** Quantification of fluorescence intensity of GFAP and phosphacan in panel A. Mean ± SD, n = 3. ^**^P < 0.01 vs. sham group; ^##^*P* < 0.01 vs. I/R group.

**Figure 5:**
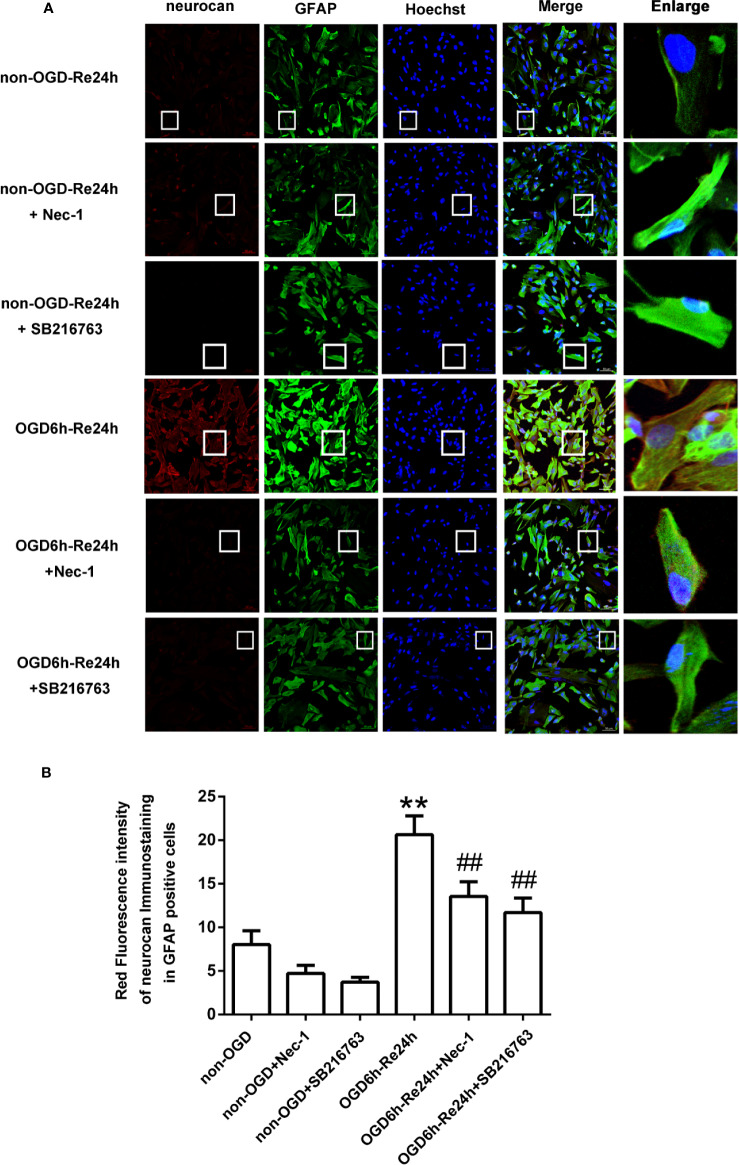
SB216763 and Nec-1 reduce the fluorescence intensity of neurocan in astrocytes. **(A)** Fluorescent double-immunostaining of glial fibrillary acidic protein (GFAP) and neurocan in primary cultured astrocytes exposed to oxygen and glucose deprivation (OGD) for 6 h and reoxygenation for 24 h after treatment with Nec-1 (100 μM) and SB216763 (5 μM) (neurocan: red; GFAP: green; Hoechst: blue). **(B)** Quantification of fluorescence intensity of neurocan in panel A. Mean ± SD, n = 3. ^**^P < 0.01 vs. non-OGD-Re24 h group; ^##^*P* < 0.01 vs. OGD6 h-Re24 h group.

**Figure 6:**
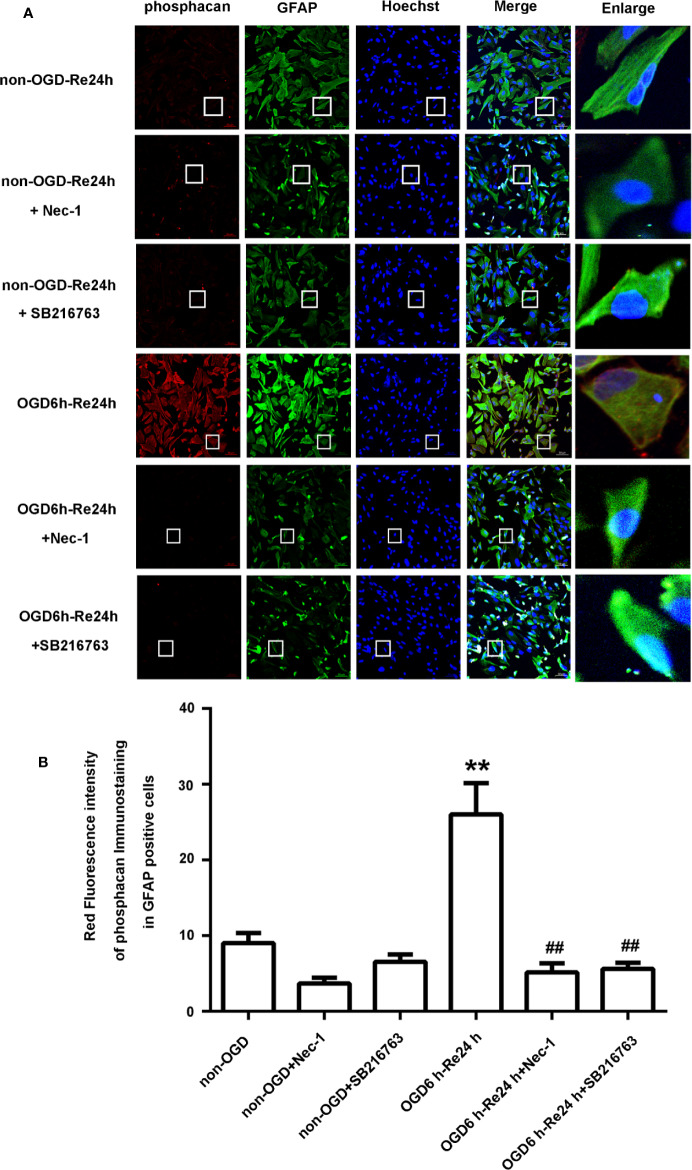
SB216763 and Nec-1 reduce the fluorescence intensity of phosphacan in astrocytes. **(A)** Fluorescent double-immunostaining of glial fibrillary acidic protein (GFAP) and phosphacan in primary cultured astrocytes exposed to oxygen and glucose deprivation (OGD) for 6 h and reoxygenation for 24 h after treatment with Nec-1 (100 μM) and SB216763 (5 μM) (phosphacan: red; GFAP: green; Hoechst: blue). **(B)** Quantification of fluorescence intensity of phosphacan in panel A. Mean ± SD, n = 3. ^**^P < 0.05 vs. non-OGD-Re24 h group; ^##^*P* < 0.01 vs. OGD6 h-Re24 h group.

A glial scar is a molecular and physical barrier produced by astrocyte proliferation during the recovery period following cerebral ischemia, which can inhibit axonal regeneration (Roll and Faissner, 2014). The protective effect of SB216763 against stroke has been reported in many studies (Rana and Singh, 2018), but its effect on glial scar formation is not well understood. Therefore, we first investigated the effect of SB216763 on the formation of glial scars induced by brain I/R injury or OGD/re-induced astrocyte injury. SB216763 was administrated *in vivo* intracerebroventricularly at 400 pmol, 10 min before MCAO. The results showed that SB216763 reduced the levels of the glial scar-related proteins such as GFAP (**Figure 2A**), neurocan (**Figure 2B**), and phosphacan (**Figure 2C**). In addition, immunohistochemistry results showed that the fluorescence intensity of the above glial scar-related proteins were significantly decreased with SB216763 treatment after I/R (**Figures 3** and **4**). *In vitro*, SB216763 was applied at 5 μM during OGD and reoxygenation. The results showed that SB216763 reduced the expression of GFAP (**Figure 2D**), neurocan (**Figure 2E**), and phosphacan (**Figure 2F**). In addition, the immunofluorescence results showed that SB216763 significantly attenuated the fluorescence intensity of the above glial scar-related proteins (**Figures 5** and **6**).

**Figure 7:**
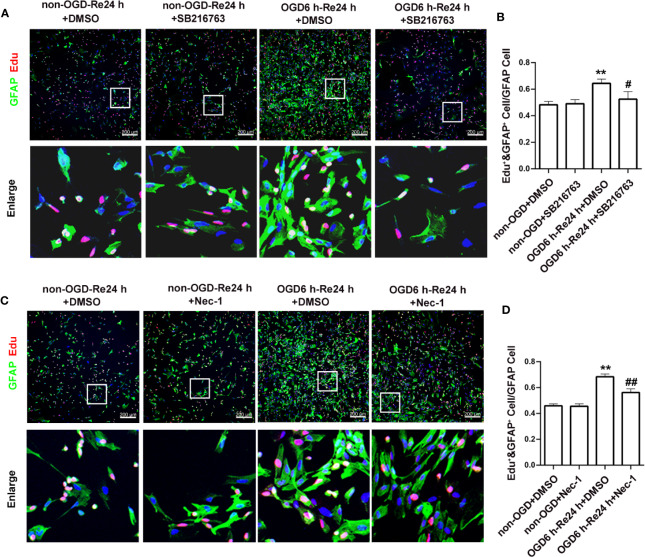
SB216763 and Nec-1 inhibit astrocyte proliferation. **(A, C)**. Astrocyte proliferation was measured by double-staining with EdU and glial fibrillary acidic protein (GFAP). The proportion of EdU-positive cells was significantly reduced after SB216763 and Nec-1 treatment (EdU: red; GFAP: green; Hoechst: blue). **(B, D)**. Quantification of fluorescence intensity of EdU and GFAP double-positive cells in panels A and B. Mean ± SD, n = 3. ^**^P < 0.05 vs. non-OGD-Re24 h+DMSO group; ^#^*P* < 0.05, ^##^*P* < 0.01 vs. OGD6 h-Re24 h+DMSO group.

Furthermore, astrocyte proliferation was determined by EdU and GFAP double-staining, with the results showing that SB216763 reduced the number of EdU and GFAP double-positive cells following OGD/Re injury (**Figures 7A, B**).

### Nec-1 Reduces Ischemic Stroke-Induced Astrogliosis *In Vivo* and *In Vitro*

**Figure 8:**
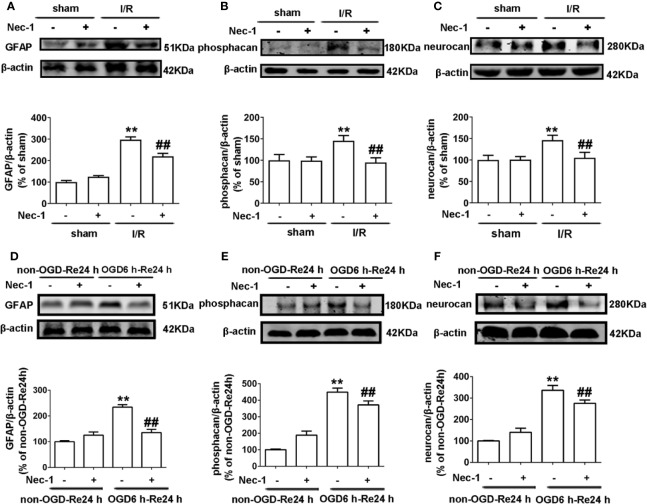
Nec-1 decreases the expression of glial scar-related proteins after OGD 6 h-Re 24 h and ischemia/reperfusion (I/R) injury. **(A–C)** Representative images from Western blotting (WB) analysis of the expression of glial fibrillary acidic protein (GFAP), neurocan, and phosphacan in the peri-infarct region of sham or cerebral ischemic cortex at 7 d after reperfusion, following I/R for 90 min. Columns present data from the quantitative analysis of GFAP, neurocan, and phosphacan immunoblots respectively. Nec-1 (48 nmol) was intracerebroventricularly administrated before ischemia. β-Actin protein was used as a loading control. Mean ± SD, n = 3. ^**^P < 0.01 vs. sham group; ^##^*P* < 0.01 vs. I/R group. **(D–F)** Representative images from Western blotting (WB) analysis of GFAP, neurocan, and phosphacan in astrocytes exposed to OGD for 6 h and reoxygenation for 24 h. Astrocytes were treated with Nec-1 (100 μM) during OGD and reoxygenation. β-Actin protein was used as a loading control. Mean ± SD, n = 3. ^**^P < 0.01, vs. non-OGD-Re24 h group; ^##^*P* < 0.01 vs. OGD6 h-Re24 h group.

We previously reported that Nec-1 protects astrocytes against pMCAO-induced injury (Ni et al., 2018). However, the role of Nec-1 in glial scar formation is unknown. Therefore, we next observed the effect of Nec-1 on glial scar formation induced by I/R injury *in vivo*. Nec-1 was administered intracerebroventricularly at 48 nmol at 10 min before MCAO and samples were taken in the peri-infarct region at 7 d after I/R injury. The results showed that Nec-1 reduced the expression of the glial scar-related proteins including GFAP (**Figure 8A**), phosphacan (**Figure 8B**), and neurocan (**Figure 8C**). In addition, the immunohistochemistry results showed that the fluorescence intensity of glial scar-related proteins such as GFAP, neurocan, and phosphacan was significantly decreased with Nec-1 treatment after I/R (**Figures 3** and **4**). *In vitro*, Nec-1 was applied at the optimum concentration of 100 μM. The results showed that Nec-1 treatment reduced the expression of GFAP (**Figure 8D**), phosphacan (**Figure 8E**), and neurocan (**Figure 8F**). The immunocytochemical results further demonstrated that Nec-1 application significantly attenuated the fluorescence intensity of GFAP, neurocan, and phosphacan (**Figures 5** and **6**).

Furthermore, astrocyte proliferation was determined by EdU and GFAP double-staining, and the results showed that Nec-1 reduced the number of EdU and GFAP double-positive cells following OGD/Re injury (**Figures 7C, D**).

### SB216763 and Nec-1 Reduce Levels of Inflammatory-Related Cytokines in Astrocytes

**Figure 9:**
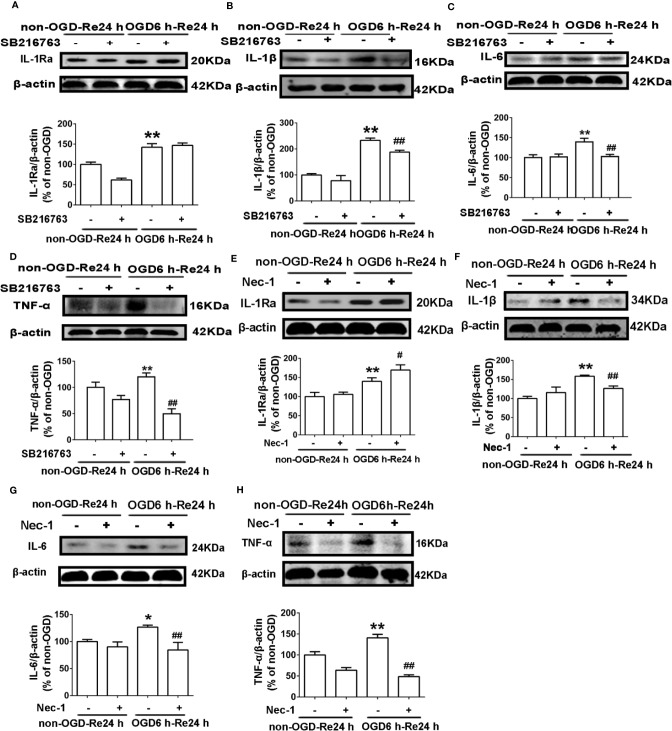
Nec-1 and SB216763 reduce the levels of inflammatory-related cytokines in astrocytes after OGD6 h-Re24 h. **(A–D)** Representative images from Western blotting (WB) analysis of IL-1Ra, IL-1β, IL-6, and TNF-α in astrocytes. Astrocytes were treated with or without SB216763 (5 μM) during OGD and reoxygenation. β-Actin protein was used as a loading control. Mean ± SD, n = 3. ^**^P < 0.01, vs. non-OGD-Re24 h group; ^##^*P* < 0.01 vs. OGD6 h-Re24 h group. **(E–H)** Representative images from Western blotting (WB) analysis of IL-1Ra, IL-1β, IL-6, and TNF-α. Astrocytes were exposed to OGD for 6 h and reoxygenation for 24 h. Astrocytes were treated with or without Nec-1 (100 μM) during OGD and reoxygenation. β-Actin protein was used as a loading control. Mean ± SD, n = 3. ^*^P < 0.05, ^**^P < 0.01 vs. non-OGD-Re24 h group; ^#^*P* < 0.05, ^##^*P* < 0.01 vs. OGD6 h-Re24 h group.

The above results showed that SB216763 and Nec-1 reduce the formation of glial scars *in vivo* and *in vitro*. Glial scar formation inhibits axonal regeneration and produces a large number of inflammatory cytokines which adhere to cells in the region of glial scar (Rolls et al., 2009). Therefore, to next explore whether SB216763 and Nec-1 reduce glial scar formation by inhibiting the inflammation, we measured levels of inflammatory-related cytokines, including IL-6, IL-1β, TNF-α, and IL-1Ra. The results showed that both SB216763 and Nec-1 suppressed levels of inflammatory cytokines in astrocytes, including IL-1β (**Figures 9B, F**), IL-6 (**Figures 9C, G**), and TNF-α (**Figures 9D, H**), but only Nec-1 increased the level of IL-1Ra (**Figures 9A, E**).

### Neutralizing Inflammatory-Related Cytokine Reduces Ischemic Stroke-Induced Astrogliosis

**Figure 10:**
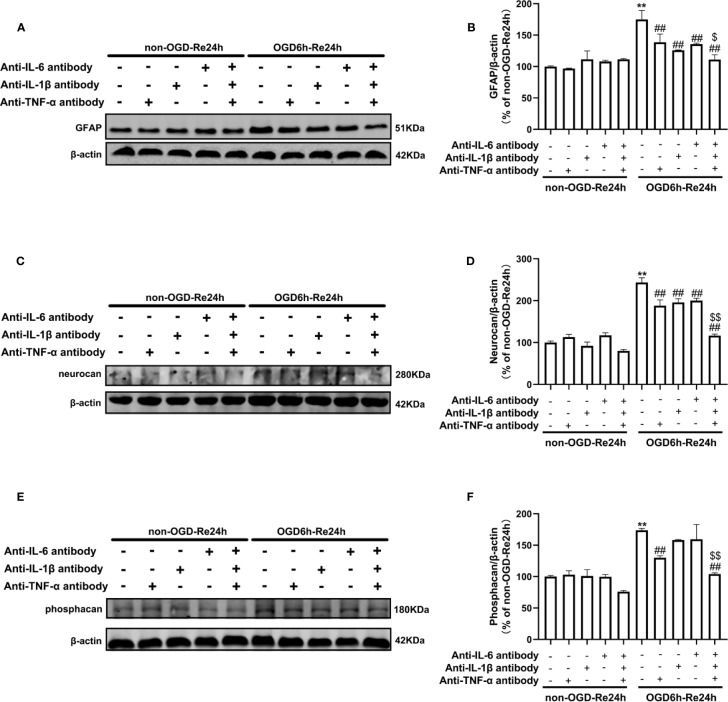
Neutralizing inflammatory-related cytokine reduces the levels of glial scar-related proteins after OGD6/Re. Astrocytes were exposed to OGD for 6 h and reoxygenation for 24 h. Astrocytes were treated with or without anti-TNF-α antibody (10 μg/ml), anti-IL-1β antibody (10 μg/ml), anti-IL-6 antibody (10 μg/ml), or anti-TNF-α antibody (10 μg/ml) + anti-IL-1β antibody (10 μg/ml) + anti-IL-6 antibody (10 μg/ml) during reoxygenation. **(A, C**, **E)** Representative images of Western blotting (WB) analysis of glial fibrillary acidic protein (GFAP), neurocan, and phosphacan. **(B, D**, **F)** Columns present data of quantitative analysis in panel A–C. Mean ± SD, n = 3. ^**^P < 0.01, vs. non-OGD-Re24 h group; ^##^*P* < 0.01 vs. OGD6 h-Re24 h group. ^$^*P*< 0.05, ^$$^*P*< 0.01, vs. OGD 6h-Re24h + anti-TNF-α antibody group, or vs. OGD6h-Re24h + anti-IL-1β antibody group or vs. OGD6h-Re24h + anti-IL-6 antibody group.

The above results showed that both SB216763 and Nec-1 suppressed levels of inflammatory cytokines in astrocytes. Therefore, we propose that SB216763 and Nec-1 reduces ischemic stroke-induced astrogliosis may be related to its inhibitory effect on inflammation. In order to verify our hypothesis, we used antibodies to neutralize the cytokines of TNF-α, IL-1β, or IL-6, and detected the glial scar-like changes in astrocytes after OGD/Re. Expectedly, we found that neutralizing of TNF-α, IL-1β, or IL-6 with each of their antibodies reduced the levels of the glial scar makers such as GFAP, neurocan, and phosphacan, further concurrent neutralization of TNF-α, IL-1β, or IL-6 with their antibodies provided better reduction in OGD/re-induced increases in glial scar markers than obtained with separate use of individual antibody (**Figure 10**).

### SB216763 and Nec-1 Inhibit the Activity of GSK3β and RIP1K

**Figure 11:**
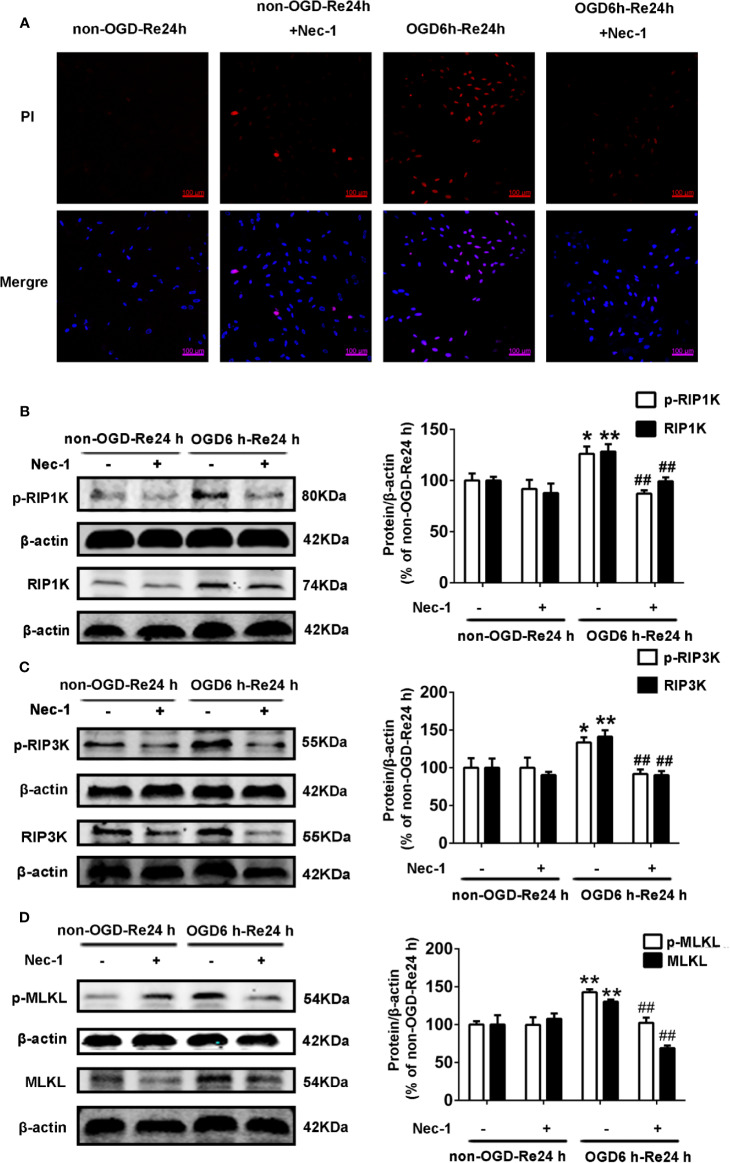
Nec-1 inhibits the levels of necroptosis-related proteins and PI-positive cells in astrocytes. Astrocytes were exposed to oxygen and glucose deprivation (OGD) for 6 h followed by reoxygenation for 24 h. **(A)** Astrocytes were treated with Nec-1 (100 μM) during OGD and OGD/Re. Cell death was assessed with propidium iodide (PI) and Hoechst staining (PI: red; Hoechst: blue). **(B–D)** Representative images from Western blotting (WB) analysis of the levels of RIP1K, p-RIP1K, RIP3K, p-RIP3K, MLKL, and p-MLKL in astrocytes. β-Actin protein was used as a loading control. Mean ± SD, n = 3. ^**^P < 0.01, ^*^P < 0.05 vs. non-OGD-Re24 h group; ^##^*P* < 0.01 vs. OGD6 h-Re24 h group.

**Figure 12:**
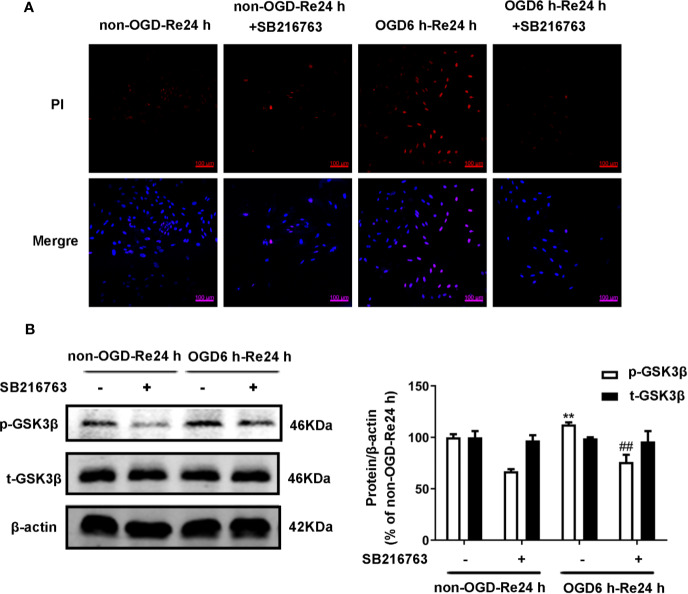
SB216763 inhibits the activity of GSK3β. Astrocytes were exposed to OGD for 6 h and reoxygenation for 24 h. **(A)** Astrocytes were treated with SB216763 (5 μM) during OGD and reoxygenation. Cell death was assessed with propidium iodide (PI) and Hoechst staining (PI: red; Hoechst: blue). **(B)** Representative images from Western blotting (WB) analysis of the levels of GSK3β-related proteins in astrocytes. β-Actin protein was used as a loading control. Mean ± SD, n = 3. ^**^P < 0.01 vs. non-OGD-Re24 h group; ^##^*P* < 0.01 vs. OGD6 h-Re24 h group.

Firstly, we measured the effects of SB216763 and Nec-1 on the kinase activities of GSK3β and RIP1K. We found that Nec-1 reduced the number of PI-positive cells (**Figure 11A**) and the expression of the necroptosis-related proteins RIP1K, p-RIP1K, RIP3K, p-RIP3K, MLKL, and p-MLKL (**Figures 11B–D**). SB216763 also reduced the number of PI-positive cells (**Figure 12A**) and the expression of p-GSK3β (**Figure 12B**).

### Interaction of GSK3β and RIP1K in the Formation of a Glial Scar

**Figure 13:**
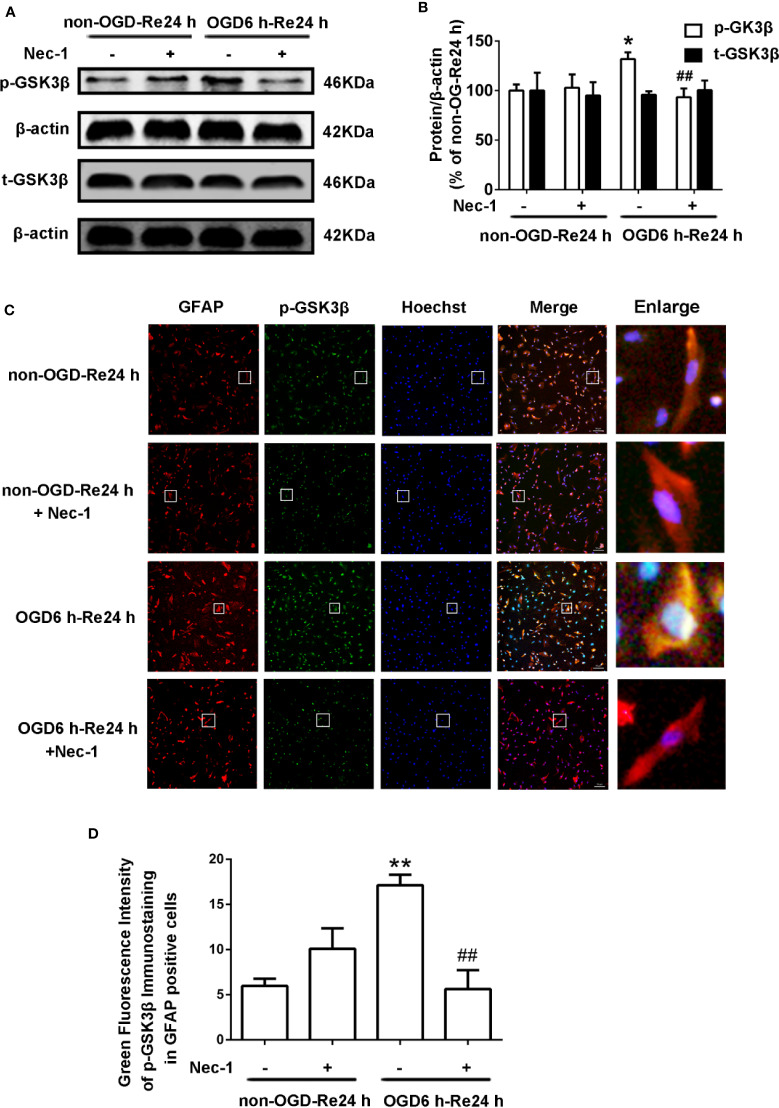
Nec-1 reduces the levels of p-GSK3β in astrocytes exposed to OGD for 6 h and reoxygenation for 24 h. Astrocytes were treated with or without Nec-1 (100 μM) during OGD and reoxygenation. **(A, B)** Representative images from Western blotting (WB) analysis of the p-GSK3β and t-GSK3β. Columns present data from the quantitative analysis of p-GSK3β and t-GSK3β immunoblots, respectively. β-Actin protein was used as a loading control. Mean ± SD, n = 3. ^*^P < 0.05 vs. non-OGD-Re24 h group; ^##^*P* < 0.01 vs. OGD6 h-Re24 h group. **(C)** Fluorescent double-immunostaining of glial fibrillary acidic protein (GFAP) and p-GSK3β in primary cultured astrocytes (GFAP: red; p-GSK3β: green; Hoechst: blue). **(D)** Quantification of fluorescence intensity of p-GSK3β immunostaining in panel C. Mean ± SD, n = 3. ^**^P < 0.01 vs. non-OGD-Re24 h group; ^##^*P* < 0.01 vs. OGD6 h-Re24 h group.

**Figure 14:**
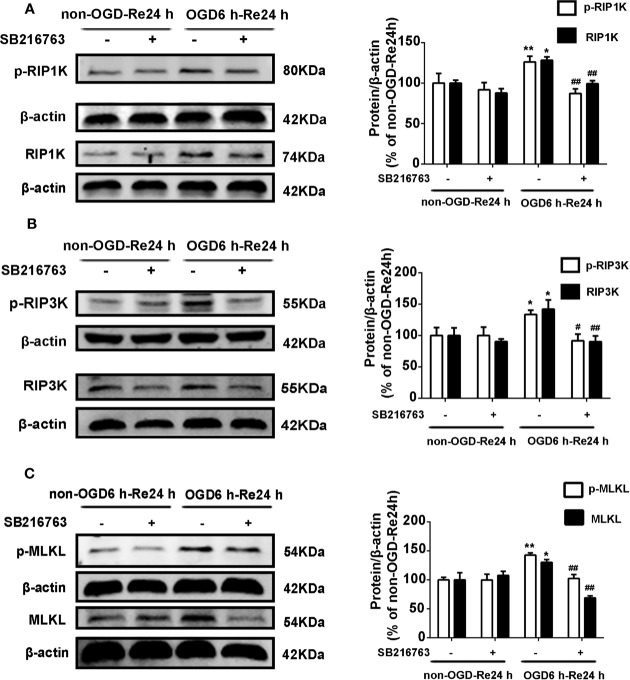
SB216763 reduces the levels of necroptosis-related proteins in astrocytes. Astrocytes were exposed to OGD for 6 h and reoxygenation for 24 h. Astrocytes were treated with or without SB216763 (5 μM) during OGD and reoxygenation. **(A–C)** Representative images from Western blotting (WB) analysis of the levels of RIP1K, p-RIP1K, RIP3K, p-RIP3K, MLKL, and p-MLKL. Columns present data from the quantitative analysis of RIP1K, p-RIP1K, RIP3K, p-RIP3K, MLKL, and p-MLKL immunoblots, respectively. β-Actin protein was used as a loading control. Mean ± SD, n = 3. ^**^P < 0.01, ^*^P < 0.05 vs. non-OGD-Re24 h group; ^##^*P* < 0.01, ^#^*P* < 0.05 vs. OGD6 h-Re24 h group.

**Figure 15:**
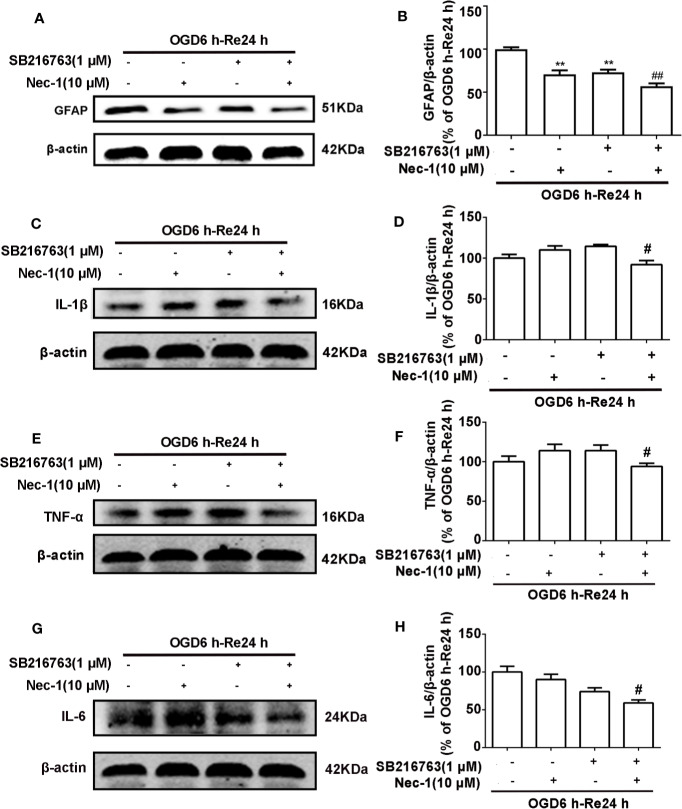
Combination of necrostatin-1 (Nec-1) and SB216763 reduces levels of glial fibrillary acidic protein (GFAP) and inflammatory cytokines. Astrocytes were exposed to OGD for 6 h and reoxygenation for 24 h. Astrocytes were treated with SB216763 (1 μM) or Nec-1 (10 μM) alone or a combination of Nec-1 and SB216763 during OGD and reoxygenation. **(A, C, E, G)** Representative images from Western blotting (WB) analysis of the levels of GFAP, IL-1β, TNF-α, and IL-6. **(B, D, F, H)** Columns present data from the quantitative analysis of GFAP, IL-1β, TNF-α, and IL-6 immunoblots in panels A, C, E, and G. β-Actin protein was used as a loading control. Mean ± SD, n = 3. ^#^*P* < 0.05, ^##^*P* < 0.01 vs. OGD6 h-Re24 h+Nec-1 group and OGD6 h-Re24 h+SB216763 group, ^**^P < 0.01 vs. OGD6 h-Re24 h group.

The relationship between GSK3β and RIP1K is not currently understood, but it has been reported that RIP1K is the key kinase responsible for activating Akt *via* the lipopolysaccharide (LPS)-induced toll-like receptor 4 (toll-like 4) signaling pathway (Vivarelli et al., 2004), and that GSK3β is an important downstream protein target of Akt. Therefore, we next explored the interaction between GSK3β and RIP1K during ischemic stroke-induced glial scar formation. The results showed that Nec-1 reduced levels of p-GSK3β (**Figures 13A, B**) and the fluorescence intensity of p-GSK3β in reactive astrocytes (**Figures 13C, D**). On the other hand, SB216763 reduced the expression of necroptosis-related proteins RIP1K, p-RIP1K, RIP3K, p-RIP3K, MLKL, and p-MLKL (**Figures 14A–C**). Based on these results, we surmised that the combination of Nec-1 and SB216763 would boost the reduction in the glial scar formation. We therefore added both inhibitors and measured the levels of glial scar-related proteins and inflammatory cytokines. The results showed that the combination of Nec-1 (10 μM) and SB216763 (1 μM) did indeed boost the reduction of GFAP level (**Figure 15A**), as well as those of inflammatory cytokines, including IL-6, IL-1β, and TNF-α, compared with Nec-1 (10 μM) or SB216763 (1 μM) alone (**Figures 15B, C**). These data indicated that the combination of lower dose of Nec-1 (10 uM) and SB216763 (1 uM) might produce synergistic pharmacological effects in reducing the glial scar formation.

## Discussion

The glial scar is a biochemical and mechanical barrier which can inhibit axonal regeneration and produce a large number of inflammatory cytokines in the chronic stage following ischemic stroke (Silver and Miller, 2004; Cai et al., 2017). Necroptosis is a form of regulated necrotic cell death, in which RIPK1/RIPK3 kinases mediate activation of the MLKL kinase (Rodriguez et al., 2016). RIP1K can activate the PI3K-Akt pathway, in which GSK3β is an important downstream protein target (Ko et al., 2015). Both inhibition of RIP1K by Nec-1 and inhibition of GSK3β by SB216763 produce a protective effect against ischemic stroke (Valerio et al., 2011; Wang et al., 2019). Here, we present a couple of novel findings: (1) both SB216763 and Nec-1 attenuate ischemic stroke-induced glial scar formation *via* inhibition of inflammatory cytokines in astrocytes; (2) SB216763 and Nec-1 attenuate inflammatory cytokines by blocking RIP1K or GSK3β activation, and by reducing an interaction between RIP1K and GSK3β during glial scar formation. (3) A combination of lower dose of Nec-1 (10 uM) and SB216763 (1 uM) might produce synergistic pharmacological effects in reducing the glial scar formation.

Previous results from our laboratory have shown that glial scar formation in the peri-infarct area of the cerebral cortex is most pronounced after MCAO for 90 min following reperfusion for 7 d in rats, and in primary cultured astrocytes after OGD for 6 h following reoxygenation for 24 h (Zhu et al., 2017). Therefore, we chose to use these *in vivo* and *in vitro* models in the present study. We found that both SB216763 and Nec-1 attenuated the glial scar-related proteins including GFAP, neurocan, and phosphacan. In order to further demonstrate the effects of Nec-1 and SB216763 on astrocyte proliferation, we performed double-staining with EdU and GFAP. The results demonstrated that application of SB216763 or Nec-1 did indeed inhibit astrocyte proliferation.

What is the mechanism by which Nec-1 and SB216763 reduce glial scar formation? In the chronic stage following ischemic stroke, a large number of inflammatory cells are recruited to the glial scar, and astrocytes in the peri-infarct region trigger the inflammatory response and exert a strong pro-inflammatory effect (Chen et al., 2018a).

It has been reported that GSK3β plays a critical role in the inflammatory response in multiple cells including astrocytes and microglia. GSK-3β inhibitors suppressed inflammatory response in 6-OHDA-activated astrocytes or in autophagy deficiency microglia (Zhou et al., 2011; Wang et al., 2013). RIP1 kinase signaling plays crucial roles in mediating necroptosis and inflammation in cellular models of necroptosis. In mice SCI, reactive astrocytes died by RIP3/MLKL-mediated necroptosis, rather than apoptosis or autophagy; suppressing RIP1 by Nec-1 or knockout of RIP3 not only reduced astrocyte death but also rescued the neurotrophic function of astrocytes (Fan et al., 2016). In zVAD.fmk-induced necroptosis of L929 cells or J774 cells, a mouse macrophage cell line, Nec-1 treatment could inhibit the production of TNF-α depending the kinase activity of RIP1 (Christofferson et al., 2012). In agreement with these recent reports, our results showed that both SB216763 and Nec-1 attenuated levels of inflammatory-related cytokines released from astrocytes, including IL-6, IL-1β, and TNF-α. In addition, Nec-1 also increased the levels of IL-1Ra. Furthermore, the current study provided evidences that reducing astrocyte-mediated inflammatory response contributed to attenuating ischemic stroke-induced glial scar formation. In the OGD/re-induced glial scar changes in astrocytes, we used antibodies to neutralize the cytokines including TNF-α, IL-1β, and IL-6 when reoxygenation, and we found that neutralizing of TNF-α, IL-1β, or IL-6 with their antibodies did reduce the levels of the glial scar makers, and concurrent neutralization of TNF-α, IL-1β, or IL-6 with their antibodies provided better reduction in OGD/re-induced increases in scar markers than obtained with separate use of each antibody. These data indicate that the SB216763- and Nec-1-mediated reductions in glial scar formation might be associated with an inhibition of inflammatory cytokine production in astrocytes in the late stage of ischemic stroke. However, as both Nec-1 and SB216763 reduce infarct size in the acute stage of ischemia in animals subjected to tMCAO- or pMCAO-induced injury (Valerio et al., 2011; Chen et al., 2018b; Ni et al., 2018; Wang et al., 2019), we cannot exclude the potential contribution of Nec-1 or SB216763 in mediating a reduction in infarct size. In addition, based on the fact that activation of resident brain cells (mainly microglia) and various types of inflammatory cells (including neutrophils, different subtypes of T cells, monocyte/macrophages, and other cells) infiltrated into the ischemic brain tissue are involved in the ischemia/reperfusion-induced inflammatory process (Jin et al., 2010), we cannot rule out the possibility that these inhibitors might also act on other surrounding immune cells, such as microglial and macrophage following ischemia/reperfusion injury.

One interesting finding from the present study is that GSK3β and RIP1K may interact to regulate ischemic stroke-induced glial scar formation. GSK3β activity is sensitive to serum deprivation, hypoxia, endotoxin exposure, and tissue hypoxia (Gall et al., 2011). The level of p-GSK3β has been reported to increase in neurons exposed to OGD for 1 h, followed by reoxygenation for 24 h, or in TNF/z-VAD-treated HT22 cells, and Nec-1 treatment reduced the increased level of TNF/zVAD-induced p-GSK3β in HT22 cells (Liu et al., 2014). Phospho-RIP1K binds to Phospho-RIP3K to form necrosome in programmed necrosis (Li et al., 2012). In our previous study (Ni et al., 2018), we used co-immunoprecipitation analysis to demonstrate that RIP1K combined with RIP3K to form a RIP1K–RIP3K necrosome after OGD, and Nec-1 could reduce the formation of this necrosome, indicating that OGD induce the activation of RIP1K and Nec-1 can inhibit the activation of RIP1K in our experiment systems. In the current study, we examined astrocytes exposed to OGD/Re injury and found that Nec-1 treatment not only decreased the levels of necroptosis-related kinases, such as RIP1K/p-RIP1K, RIP3K/p-RIP3K, and MLKL/p-MLKL, but also of p-GSK3β. Conversely, SB216763 not only inhibited the level of p-GSK3β, but also reduced levels of RIP1K/p-RIP1K, RIP3K/p-RIP3K, and MLKL/p-MLKL. Moreover, Nec-1 and/or SB216763 inhibited the LDH leakage and reduced numbers of PI-positive cells, demonstrating the protective effect of Nec-1 and SB216763 on OGD/re-induced astrocytic cell necroptosis. Furthermore, compared with lower dose of Nec-1 (10 μM) and SB216763 (1 μM) alone, application of the two inhibitors together reduced the levels of glial scar-related markers such as GFAP, neurocan, and inflammatory-related cytokines, such as IL-6, TNF-α, and IL-1β. These data indicate that SB216763 and Nec-1 inhibit inflammatory cytokine production by blocking RIP1K and GSK3β activation, and that these two kinases may interact during the formation of ischemic stroke-induced glial scars; the combination of lower dose of Nec-1 (10 uM) and SB216763 (1 uM) might produce synergistic pharmacological effects in reducing the glial scar formation. It has been found that introduction of adenoviral RIP1 into RIP1^−/−^ MEFs resulted in a significant phosphorylation of Akt and phosphorylation of GSK3 (Park et al., 2008), whereas, the phosphoinositide 3-kinase (PI3K) signaling pathway is responsible for the inhibition of GSK-3 by mediating its phosphorylation (Maurer et al., 2014). Thus, whether RIP1K interacts with GSK3β directly or *via* phosphor-Akt in astrocytes remains further investigated in the very near future.

In conclusion, our data show that Nec-1 and SB216763 contribute to reducing glial scar formation following OGD/re- and I/R-induced injury, at least, partially associating with suppressing astrocyte-mediated production of inflammatory cytokines and with blocking the activation and an interaction of RIP1K and GSK3β. Lower dose of Nec-1 and SB216763 boost the inhibitory effect on glial scar formation. Our work provides additional evidence demonstrating the crucial benefit that Nec-1 and SB216763 can bring during the chronic stage of ischemic stroke.

## Data Availability Statement

All datasets generated for this study are included in the article/supplementary material.

## Ethics Statement

The animal study was reviewed and approved by Ethics Committee of Soochow University (ECSU). Written informed consent was obtained from the owners for the participation of their animals in this study.

## Author Contributions

H-LZ designed the research and provided funding; JL, Y-MZ, YG, LL, FG, X-YD, MZ, and Y-FW carried out experiments; JL, LL and Y-MZ analyzed experimental results. JL and Y-MZ wrote the manuscript, and H-LZ and Z-XW revised it.

## Funding

This work was supported by grants from the National Natural Science Foundation of China (Nos. 81874311, 81171104, and 81473211), the Jiangsu Key Laboratory of Neuropsychiatric Diseases (No. BM2013003), the Jiangsu Key Laboratory of Preventive and Translational Medicine for Geriatric Diseases, The Postgraduate Research & Practice Innovation Program of Jiangsu Province (No. KYCX18_2542), and the funding from the Priority Academic Program Development (PAPD) of Jiangsu Higher Education Institutions.

## Conflict of Interest

The authors declare that the research was conducted in the absence of any commercial or financial relationships that could be construed as a potential conflict of interest.

## Abbreviations

Nec-1, necrostatin-1; GSK3β, glycogen synthase kinase-3β; IL-6, interleukin-6; IL-1β, interleukin-1β; TNF-α, tumor necrosis factor-α; IL-1Ra, interleukin-1 receptor antagonist; tMCAO, transient middle cerebral artery occlusion; I/R, ischemia/reperfusion; OGD/Re, oxygen and glucose deprivation and reoxygenation; PI, propidium iodide; GFAP, glial fibrillary acidic protein; SD, Sprague-Dawley; LDH, lactate dehydrogenase.
